# Pomegranate Peel Extract Attenuates Isoprenaline-Induced Takotsubo-like Myocardial Injury in Rats

**DOI:** 10.3390/pharmaceutics15061697

**Published:** 2023-06-09

**Authors:** Sonja T. Marinković, Đorđe Đukanović, Mladen Duran, Zorislava Bajic, Tanja Sobot, Snežana Uletilović, Nebojša Mandić-Kovacević, Tanja Cvjetković, Žana M. Maksimović, Uglješa Maličević, Nikolina Vesić, Sanja Jovičić, Maja Katana, Katarina Šavikin, Dragan M. Djuric, Miloš P. Stojiljković, Ranko Škrbić

**Affiliations:** 1 Centre for Biomedical Research, Faculty of Medicine, University of Banja Luka, 78000 Banja Luka, The Republic of Srpska, Bosnia and Herzegovina; djordje.djukanovic@med.unibl.org (Đ.Đ.); mladen.duran@med.unibl.org (M.D.); zana.maksimovic@med.unibl.org (Ž.M.M.); ugljesa.malicevic@med.unibl.org (U.M.); nikolina.vesic@med.unibl.org (N.V.); maja.katana@med.unibl.org (M.K.); milos.stojiljkovic@med.unibl.org (M.P.S.); ranko.skrbic@med.unibl.org (R.Š.); 2 Pediatric Clinic, University Clinical Centre of the Republic of Srpska, 78000 Banja Luka, The Republic of Srpska, Bosnia and Herzegovina; 3 Department of Physiology, Faculty of Medicine, University of Banja Luka, 78000 Banja Luka, The Republic of Srpska, Bosnia and Herzegovina; zorislava.bajic@med.unibl.org (Z.B.); tanja.sobot@med.unibl.org (T.S.); 4 Department of Medical Biochemistry and Chemistry, Faculty of Medicine, University of Banja Luka, 78000 Banja Luka, The Republic of Srpska, Bosnia and Herzegovina; snezana.uletilovic@med.unibl.org (S.U.); tanja.cvjetkovic@med.unibl.org (T.C.); 5 Department of Pharmacy, Faculty of Medicine, University of Banja Luka, 78000 Banja Luka, The Republic of Srpska, Bosnia and Herzegovina; nebojsa.mandic-kovacevic@med.unibl.org; 6 Department of Histology and Embryology, Faculty of Medicine, University of Banja Luka, 78000 Banja Luka, The Republic of Srpska, Bosnia and Herzegovina; sanja.jovicic@med.unibl.org; 7 Institute for Medicinal Plants Research “Dr Josif Pančić”, 11000 Belgrade, Serbia; katarina.savikin@gmail.com; 8 Institute of Medical Physiology “Richard Burian”, Faculty of Medicine, University of Belgrade, 11000 Belgrade, Serbia; dr_djuric@yahoo.com; 9 Department of Pharmacology, Toxicology and Clinical Pharmacology, Faculty of Medicine, University of Banja Luka, 78000 Banja Luka, The Republic of Srpska, Bosnia and Herzegovina

**Keywords:** isoprenaline, takotsubo cardiomyopathy, cardioprotection, *Punica granatum*, oxidative stress

## Abstract

Takotsubo syndrome (TTS) is an acute heart failure syndrome characterised by catecholamine-induced oxidative tissue damage. *Punica granatum*, a fruit-bearing tree, is known to have high polyphenolic content and has been proven to be a potent antioxidant. This study aimed to investigate the effects of pomegranate peel extract (PoPEx) pre-treatment on isoprenaline-induced takotsubo-like myocardial injury in rats. Male Wistar rats were randomised into four groups. Animals in the PoPEx(P) and PoPEx + isoprenaline group (P + I) were pre-treated for 7 days with 100 mg/kg/day of PoPEx. On the sixth and the seventh day, TTS-like syndrome was induced in rats from the isoprenaline(I) and P + I groups by administering 85 mg/kg/day of isoprenaline. PoPEx pre-treatment led to the elevation of superoxide dismutase and catalase (*p* < 0.05), reduced glutathione (*p* < 0.001) levels, decreased the thiobarbituric acid reactive substances (*p* < 0.001), H_2_O_2_, O_2_^−^ (*p* < 0.05), and NO_2_^−^ (*p* < 0.001), in the P + I group, when compared to the I group. In addition, a significant reduction in the levels of cardiac damage markers, as well as a reduction in the extent of cardiac damage, was found. In conclusion, PoPEx pre-treatment significantly attenuated the isoprenaline-induced myocardial damage, primarily via the preservation of endogenous antioxidant capacity in the rat model of takotsubo-like cardiomyopathy.

## 1. Introduction

Takotsubo syndrome (TTS) is an acute heart failure syndrome, also known as “broken heart syndrome”, that usually occurs after extreme physical and/or emotional stress [1,2]. Research has shown that TTS has similar clinical presentation and mortality rates as acute myocardial infarction (AMI) [3,4]. Reports have shown that the prevalence of TTS is approximately 2% (or up to 10% if only women are considered) of all patients with a clinical presentation of acute coronary syndrome [5]. However, underlying pathophysiological mechanisms are different. In contrast to AMI, no significant coronary artery obstruction can be found in patients with TTS [6]. Typical findings in TTS are severe regional left ventricular dysfunction, with akinesia of apical segments, that is associated with a metabolic derangement of the affected myocardium [7]. Although the exact pathophysiological mechanism is not entirely understood, the leading hypothesis of TTS pathogenesis seems to be catecholamine-induced myocardial overstimulation [8]. This correlates with reports of conditions such as phaeochromocytoma and thyrotoxicosis causing TTS [9,10], as well as with findings that both adrenaline and noradrenaline levels are notably more elevated in TTS than in AMI [11].

The isoprenaline model of myocardial injury is a long-standing animal experimental model of myocardial infarction that was first described in the early 1960s by Chappel and Rona [12,13]. The model has been widely used and investigated. In previous studies by Shao et al., a novel rat model of TTS was proposed [7]. They showed that isoprenaline administration replicates the most important characteristics of TTS, such as typical apical ballooning, ECG changes, a complete recovery of cardiac function, and characteristic histological findings. The isoprenaline overstimulation model has also been characterised as a suitable model of TTS by several authors [4,14] In addition, Fineschi et al. proposed a link between oxidative stress and catecholamine-induced myotoxicity [15]. Excessive catecholamines may be auto-oxidised into reactive intermediates, further leading to accumulated intracellular lipids’ peroxidation [7]. Moreover, increased ROS levels have been found in samples collected by endomyocardial biopsy from patients with takotsubo syndrome [16].

The pomegranate (*Punica granatum* L.) is a fruit-bearing tree that originates from the area of the Middle East [17]. Today, it is cultivated and consumed worldwide. The arils of pomegranate have a recognisable deep red colour due to its high polyphenolic content [18], which makes it a more potent antioxidant than vitamins E, A, and C [19]. Although arils in the form of juice are the most consumed part of the plant, pomegranate peel comprises up to 40% of the total fruit weight, and previous research has shown that it also has high polyphenolic content of 48 different phenolic compounds [20], with a particularly high content of hydrosoluble tannins, including punicalagin, punicalin, gallic acid, and ellagic acid. These polyphenols have been proven to have antioxidant, lipid-lowering, anti-inflammatory, and antihypertensive properties [17,21,22,23].

Although progress has been made in identifying causative factors for Takotsubo syndrome, treatment of TTS is still based on expert opinion and symptomatic therapy such as beta-blockers [24]. Considering that an increase in reactive oxygen species (ROS) accumulation and subsequent increase of inflammatory cells and factors can be seen both in patients suffering from TTS and in animal models [16,25], a hypothesis was derived that modulators of inflammation and antioxidants could have the potential to be a preventive or therapeutic option. Several studies have investigated the cardioprotective effects of pomegranate juice or seed juice extract in the isoprenaline model [26,27], but there are no sufficient data considering the cardioprotective potential of pomegranate peel extract (PoPEx). Therefore, this study aimed to investigate the effects of pomegranate peel extract pre-treatment on isoprenaline-induced takotsubo-like myocardial injury in rats.

## 2. Materials and Methods

### 2.1. Pomegranate Peel Extract and Isoprenaline

The pomegranate peel extract used in this study was provided by the Institute for Medicinal Plant Research “Dr Josif Pančić” (Belgrade, Serbia). Pomegranate fruits were harvested from the East Herzegovina region, the Republic of Srpska, Bosnia, and Herzegovina. After separation from the fruit, the peel parts were dried at room temperature for 4–6 days and then ground to obtain the powder. Powder (100 g) was extracted in an ultrasonic bath with 50% ethanol using a 1:10 solid-to-solvent ratio and then evaporated to dryness [28]. Its polyphenolic content was quantified using HPLC. The analysis showed that the main polyphenols were two punicalagin isomers: α- and β-punicalagin (26.02 and 45.57 mg/g dry weight (DW), respectively). The other ellagitannins in the extract were punicalin (31.31 mg/g DW), ellagic acid (22.82 mg/g DW), and gallic acid (7.74 mg/g DW) [29]. Isoprenaline hydrochloride was purchased from Sigma-Aldrich (St. Louis, MI, USA; purity > 98.5%) and dissolved using normal saline to a concentration of 85 mg/mL to achieve a dose of 1 mL/kg.

### 2.2. Chemicals

The chemicals used for the oxidative stress assays were thiobarbituric acid (Carlo Erba, Val de Reuil, France, CAS 504-17-6), sodium hydroxide (Lachner, Neratovice, Czech Republic, CAS 1310-73-2), (Ethylenedinitrilo)tetraacetic acid disodium salt (Lachner, Czech Republic, CAS 6381-92-6), sulfanilic acid (Acros Organics, Geel, Belgium, CAS 121-57-3), n-1-naphthyl ethylenediamine dihydrochloride (Fisher Chemicals, Loughborough, UK, CAS 1465-25-4), sodium chloride (Lachner, Czech Republic, CAS 7647-14-5), gelatine (Acros Organics, CAS 9000-70-8), nitrotetrazolium blue chloride (Acros Organics, CAS 298-83-9), horseradish peroxidase (Sigma Aldrich, St. Louis, MO, USA, CAS 9003-99-0), Tris(hydroxymethyl)aminomethane (Acros Organics, CAS 77-86-1), Potassium dihydrogen phosphate (Lachner, Czech Republic, CAS 7778-77-0), glucose (Lachner, Czech Republic, CAS 50-99-7), phenol red (Acros Organics, CAS 143-74-8), metaphosphoric acid (Acros Organics, CAS 37267-86-0), di-sodium hydrogen phosphate (Carlo Erba, France, CAS 7558-79-4), 5,5-dithio-bis-(2-nitrobenzoic acid) (Sigma Aldrich, USA, CAS 69-78-3), trisodium citrate dihydrate (Fisher Chemicals, UK, CAS 6132-04-3), Glutathione reduced (Acros Organics, CAS 70-18-8), and L-Epinephrine (Sigma Aldrich, USA, CAS 51-43-4).

### 2.3. Experimental Animals and Experimental Protocol

Male Wistar albino rats (*n* = 24) weighing 210 ± 20 g were used in this experiment. Animals were kept under controlled laboratory conditions, at 21 ± 2 °C room temperature, 55 ± 5% humidity, and a 12 h light-dark cycle. They were given access to food and water ad libitum. They received a standard pellet diet purchased from the Veterinary Institute of Subotica (Subotica, Serbia). Animals were randomised into four groups. Animals in the PoPEx (P; *n* = 6) and PoPEx + isoprenaline group (P + I; *n* = 7) were pre-treated with 100 mg/kg of PoPEx suspended in 0.5% carboxy methyl cellulose (CMC), while the animals in the control (C; *n* = 5) and isoprenaline (I; *n* = 6) groups received an equivalent amount of the CMC. Pre-treatment was delivered via oral gavage for 7 days. On the sixth and the seventh day, rats in the I and P + I groups received 85 mg/kg/day of the isoprenaline solution subcutaneously (s.c.), and the C and P groups received an equivalent amount of saline. On the eighth day, animals were anaesthetised using a combination of 90 mg/kg ketamine and 10 mg/kg xylazine and then sacrificed by exsanguination, and tissue and blood samples were collected.

### 2.4. Hearth Tissue Homogenisation

After excision, rat hearts were rinsed in ice-cold normal saline and frozen at −20 °C. Later on, the tissue homogenate was prepared in ice-cold phosphate buffer (pH 7.4) using an HG-15D homogeniser (Witeg Labortechnik GmbH, Wertheim, Germany) and centrifuged at +4 °C and 1200× *g*. The supernatant was used to determine levels of TBARS, SOD, CAT, and GSH.

### 2.5. Oxidative Stress Markers

Oxidative stress status was measured in heart tissue homogenate, plasma, and erythrocyte lysate. Plasma prooxidative markers, hydrogen peroxide (H_2_O_2_), superoxide anion radical (O_2_^−^), and nitrite (NO_2_^−^) were measured using Pick and Keisari method [30], Nitro Blue Tetrazolium (NBT) reduction method [31], and Green method [32], respectively. The index of lipid peroxidation, thiobarbituric acid reactive substances (TBARS), was determined using 1% TBA and 0.05 M sodium hydroxide (NaOH) and measured at 530 nm [33]. Antioxidants in erythrocyte lysate—CAT, SOD, and GSH—were measured spectrophotometrically using Beutler methods [34,35,36].

### 2.6. Serum Cardiac Markers and Lipid Profile Measurement

The concentrations of high-sensitivity troponin I (hsTnI) and homocysteine (Hcy) were measured on Abbot Alinity ci-series by chemiluminescent microparticle immunoassay (CMIA). Additional markers of cardiac damage (AST, ALT, LDH), as well as serum lipid status, were determined.

### 2.7. Histopathological Analysis

After dissection, isolated rat hearts were fixed in 10% formalin. Afterward, the samples were moulded into blocks with paraffin wax and cut into 4 µm slices using a standard issue microtome. The slices were then stained with haematoxylin and eosin dye (H&E). Microscopic analysis of the myocardial injuries was performed, each slice was scored from 1 to 4, and an average group score was calculated. A score of 1 means that there were no pathological changes in the myocardium; 2—mild damage, with multifocal degeneration and mild inflammation infiltration or focal damage of cardiomyocytes; 3—moderate damage, with severe myofibril degeneration and/or diffuse inflammation; 4—severe damage, necrosis with diffuse inflammation.

### 2.8. Statistical Analysis

Statistical analysis was performed with IBM-SPSS Statistics version 17.0 software (SPSS, Inc., Chicago, IL, USA). The Kruskal–Wallis test was used to compare the nonparametric characteristics between the groups followed by Tukey and Bonferroni tests for post hoc analysis. Results are presented as mean ± standard error, and *p* < 0.05 was considered statistically significant.

## 3. Results

### 3.1. Effects on Oxidative Stress Markers in Serum, Erythrocyte Lysate, and Heart Tissue Homogenate

**Figure 1:**
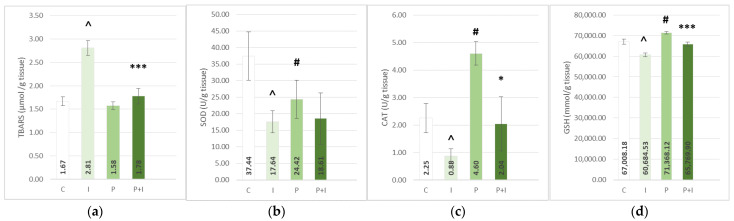
Effects of *Punica granatum* peel extract pre-treatment on heart tissue homogenate thiobarbituric acid reactive substances (TBARS) (**a**), antioxidative enzyme levels—superoxide dismutase (SOD) (**b**) and catalase (CAT) (**c**), and reduced glutathione (GSH) (**d**). All values are expressed as mean ± SEM. C—control group; P—pomegranate control group; I—isoprenaline group; P + I—pomegranate + isoprenaline group. ˄ *p* < 0.05 I vs. C; # *p* < 0.05 P vs. C; * *p* < 0.05 P + I vs. I; *** *p* < 0.001 P + I vs. I.

**Figure 2:**
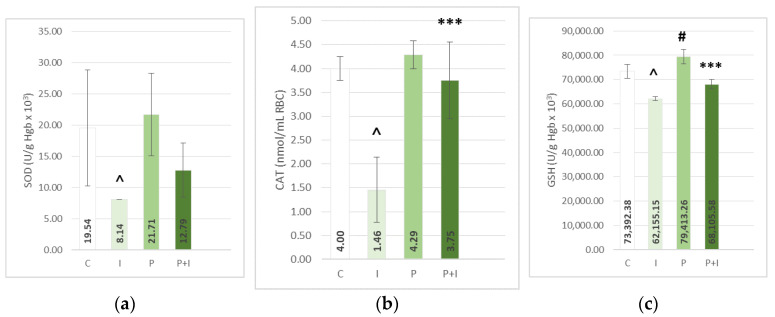
Effects of *Punica granatum* peel extract pre-treatment on the erythrocyte lysate levels of antioxidative enzymes—superoxide dismutase (SOD) (**a**) and catalase (CAT) (**b**), and reduced glutathione (GSH) (**c**). All values are expressed as mean ± SEM; C—control group; P—pomegranate control group; I—isoprenaline group; P + I—pomegranate + isoprenaline group. ˄ *p* < 0.05 I vs. C; # *p* < 0.05 P vs. C; *** *p* < 0.001 P + I vs. I.

**Figure 3:**
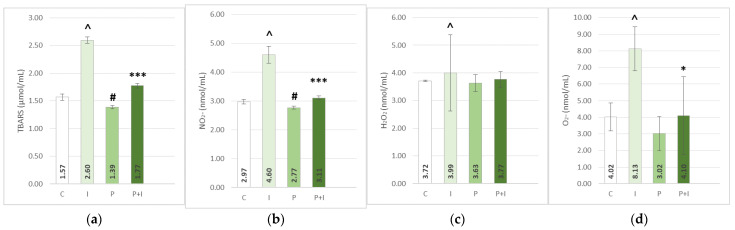
Effects of *Punica granatum* peel extract pre-treatment on plasma thiobarbituric acid reactive substances (TBARS) (**a**), NO_2_^−^ (**b**), H_2_O_2_ (**c**), and O_2_^−^ (**d**). All values are expressed as mean ± SEM. C—control group; P—pomegranate control group; I—isoprenaline group; P + I—pomegranate + isoprenaline group. ˄ *p* < 0.05 I vs. C; # *p* < 0.05 P vs. C; * *p* < 0.05 P + I vs. I; *** *p* < 0.001 P + I vs. I.

The subcutaneous application of ISO caused a significant increase in the lipid peroxidation index—thiobarbituric acid reactive substances (TBARS) in heart tissue homogenate, and a decrease in levels of antioxidative enzymes—superoxide dismutase and catalase (SOD, CAT), and reduced glutathione (GSH) measured in erythrocyte lysate and hearth tissue (Figure 1 and Figure 2). The ISO-treated groups also showed an increase in levels of plasma prooxidative markers: hydrogen peroxide (H_2_O_2_), superoxide anion radical (O_2_^−^), and nitrite (NO_2_^−^), coupled with an increase in plasma TBARS (Figure 3).

Pre-treatment with PoPEx attenuated the effects of isoprenaline and showed a significant increase in levels of antioxidative enzymes (homogenate-CAT *p* < 0.05; lysate-CAT *p* < 0.001) and GSH (GSH *p* < 0.001), as well as a decrease in prooxidative markers (O_2_^−^ *p* < 0.05; NO_2_^−^ *p* < 0.001). In addition, PoPEx-pre-treated groups showed a significant reduction of both plasma and heart tissue TBARS (*p* < 0.001). On the other hand, in the case of SOD and H_2_O_2_, pomegranate pre-treatment showed no beneficial effects. In the positive control group (P group), a significant rise of antioxidative enzymes, for example, the heart tissue GSH and CAT and lysate GSH, were found. Rats in this group also showed a significant decrease in prooxidative serum marker NO_2_^−^ and plasma TBARS. A similar pattern can be found in the case of other antioxidative enzymes, such as lysate SOD and CAT, and heart tissue TBARS and plasma H_2_O_2_ and O_2_^−^, but without statistical significance.

### 3.2. Effects on Biochemical Parameters and Serum Cardiac Markers

As the most sensitive marker of myocardial damage, hsTnI levels were determined in the collected serum samples. Results showed a significant increase (*p* < 0.01) of hsTnI levels in the I group compared to the control. This effect was significantly attenuated in the PoPEx-pre-treated (P + I) group. As additional markers of cardiac damage, the levels of AST, ALT, and LDH and the level of homocysteine were significantly increased in ISO-treated group. In the PoPEx-pre-treated (P + I) group, the serum levels of these markers were significantly lower than in the I group (Table 1). It was also noted that all the markers of cardiac damage had lower levels in the P group when compared to the control, but without statistical significance. 

### 3.3. Effects on Lipid Status

A lipid panel analysis was performed, determining levels of total cholesterol (TC), triglycerides (TG), LDL, and HDL. Although no statistical significance was found, isoprenaline administration showed a tendency toward lowering the HDL levels and rising the levels of TC, TG, and LDL (I vs. C), while PoPEx pre-treatment showed a tendency to decrease TC, TG, and LDL levels and elevate HDL levels (P + I vs. I) (Table 2).

### 3.4. Pathohistological Analyses of Rat Hearts

**Figure 4:**
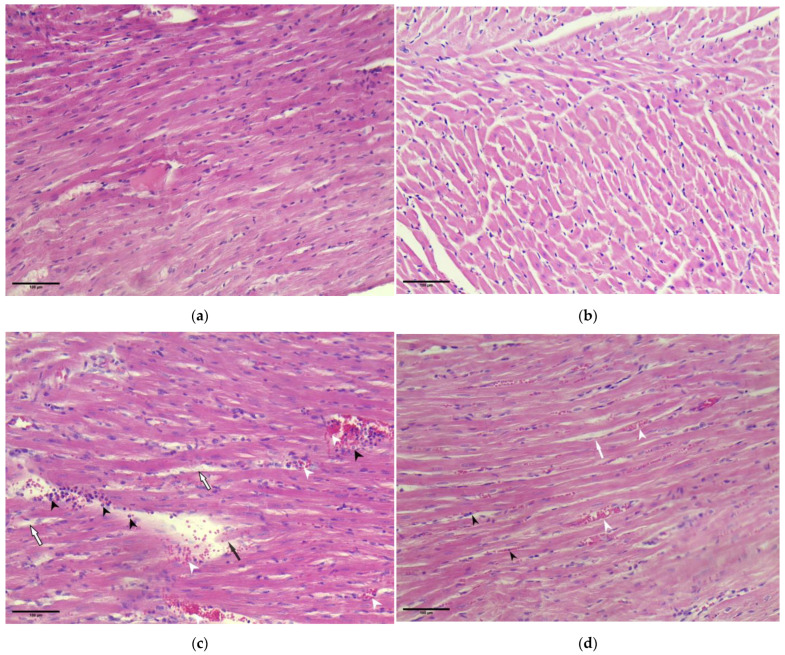
Representative microphotographs of rat heart sections stained by haematoxylin and eosin (magnification 20×, scale bar = 100 µm). Myocardium structure presenting as normal in the (**a**) control and (**b**) PoPEx group; (**c**) isoprenaline group myocardium showing severely damaged myocardium with fragmented and degenerated cardiomyocytes (black arrow), myofibril loss, interstitial oedema (white arrow), and dense inflammatory infiltrate (black arrowhead), as well as perivascular haemorrhage (white arrowhead); (**d**) PoPEx + Isoprenaline group myocardium presenting with mild damage, no degenerative cardiomyocyte changes, and a slight degree of inflammation (black arrowhead), interstitial oedema (white arrow) and haemorrhage (white arrowhead).

**Figure 5:**
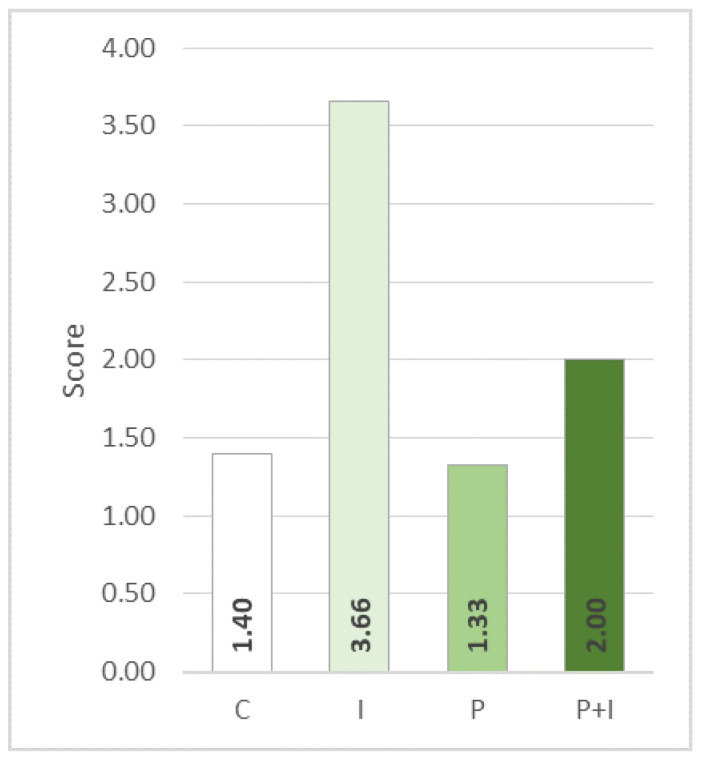
Effects of pomegranate pre-treatment on myocardial damage score. The values represent the mean damage score of the group. The following scoring system was used: Score 1—no pathological changes; Score 2—mild damage, with multifocal degeneration and mild inflammation infiltration or focal damage of cardiomyocytes; Score 3—moderate damage, with severe myofibril degeneration and/or diffuse inflammation; Score 4—severe damage, necrosis with diffuse inflammation; C—control group; P—pomegranate control group; I—isoprenaline group; P + I—pomegranate + isoprenaline group.

Microscopic investigation of myocardium samples of rats treated with isoprenaline showed severely damaged myocardium with fragmented and degenerated cardiomyocytes, loss of myofibrils, interstitial oedema, and dense inflammatory infiltrate, as well as perivascular haemorrhage. However, in the PoPEx-pre-treated (P + I) group, only a mild degree of tissue damage was found (Figure 4). The average myocardial damage score was also determined (Figure 5). A significant decrease in the level of myocardial damage was found in the P + I group when compared to the isoprenaline (I) group.

## 4. Discussion

In the present study, an isoprenaline model of takotsubo-like myocardial injury was used to investigate the cardioprotective potential of PoPEx. The results show that seven-day pre-treatment with 100 mg/kg of PoPEx led to a decrease in oxidative stress markers, an increase in the levels of antioxidant enzymes, and a reduction of myocardial damage and serum cardiac damage markers.

Isoprenaline acts as a non-selective β1,2-AR agonist via the Gs-cyclic adenosine monophosphate–protein kinase A (Gs-cyclic AMP–PKA) pathway, thus having positive chronotropic and inotropic effects on the myocardium [37]. This mimics elevated plasma catecholamines that can be found in patients with Takotsubo syndrome [11]. Previous studies have shown that isoprenaline administration in rats leads to takotsubo-like cardiomyopathy, mimicking characteristic Takotsubo syndrome findings such as apical ballooning [7]. It has been proposed that due to catecholamine overstimulation, a switch of intracellular signalling pathways, from G_s_ to G_i_ protein signalling, happens, thus causing a metabolic change in the myocardium, predominantly in the apical area, which has a higher β-AR density [7,38,39]. In addition, accumulated catecholamines are auto-oxidised, creating reactive intermediates and subsequent intracellular lipid peroxidation [7]. This makes the myocardium more susceptible to further oxidative damage, via oxidative deterioration of the membrane polyunsaturated fatty acids, which leads to the alteration of membrane structure and enzyme activity [40].

To study the extent of oxidative tissue damage and antioxidant status, levels of TBARS were measured in the collected plasma samples and heart tissue homogenate, and the activity of SOD, CAT, and GSH was determined in tissue and erythrocyte lysate samples. SOD and CAT are free radical scavenging enzymes that represent the first-line defence against oxidative tissue damage [41,42]. SOD converts superoxide radicals into hydrogen peroxide, which is then converted to molecular oxygen and water by CAT [27,43]. On the other hand, glutathione peroxidase leads to the reduction of hydrogen peroxide radicals. Consequently, the levels of all three parameters are decreased when tissues are exposed to oxidative damage due to increased utilisation, while the levels of hydrogen peroxide, superoxide anion radical, and nitrite increase. PoPEx pre-treatment caused a restoration of antioxidative enzyme levels and a decrease of the free radical levels, suggesting that pomegranate acts as a free radical scavenger, thus sparing the antioxidant capacity of endogenous enzymes. A similar result was found in a study by Jadeja et al., who used pomegranate juice as a pre-treatment [27].

Although it is understood that catecholamine overstimulation plays a major role in the pathogenesis of Takotsubo syndrome, less is known about the subcellular mechanisms of the cardiac dysfunction that follows the acute damage. Overdosing rats with isoprenaline causes injury of the myocardium, primarily in the apex area, that later undergoes cardiac remodelling and subsequent dysfunction [14]. Willis and collaborators found that mitochondrial dysfunction and exacerbated oxidative stress were causative factors of cardiac dysfunction in isoprenaline-induced Takotsubo-like cardiomyopathy [14]. This suggests that the antioxidative capacity of PoPEx is an important component of its cardioprotective potential. The antioxidative potential of pomegranate peel was previously demonstrated in both in vivo and in vitro studies, and the results were summarised in a recent review by Fahmy et al. [44]. The main reason for the high antioxidant potential of PoPEx is thought to be its high polyphenolic content. The major polyphenols in the PoPEx used in the present study were α- and β-punicalagin, followed by punicalin and gallic and ellagic acid [29]. Its antioxidant capacity was previously investigated in a study by Mandić–Kovačević et al., who used a variety of in vitro models and showed high values of antioxidant capacity [45]. Other studies have shown that among other more widely used pomegranate products, such as pomegranate pulp, seed, and juice, PoPEx has significantly higher antioxidative capacity [46,47]. Although the present study focused on providing initial evidence of the cardioprotective potential of PoPEx in experimentally induced Takotsubo-like cardiomyopathy, mechanisms by which PoPEx and/or its polyphenols exhibit their antioxidative and anti-inflammatory activities can be found. It has been shown that peel extracts have the capacity to scavenge superoxide, hydroxyl anion, and peroxyl radicals [48]. The mechanism through which polyphenols scavenge radicals is considered to be a donation of hydrogen atoms, which reduces radicals to their non-radical form, i.e., DPPH is reduced to DPPH-H. This consequently inhibits radical activity [49]. In addition, Al-Gubory et al. showed that pomegranate peel extract also acts via upregulation of the antioxidant enzymes activity, such as SOD and CAT, glutathione peroxidase (GPx), glutathione-S-transferase (GST), and glutathione reductase (GR) [50].

Mechanical damage or the destruction of myocytes due to ischaemia leads to damage or even ruptures in their cellular membranes. This results in the leakage of intracellular enzymes into the bloodstream, thus elevating their activities [40,51]. As it is known, the extent of tissue damage is proportional to the amount of enzyme released [52] Well-known markers of cardiac muscle damage, hsTnI, AST, ALT, and LDH were measured. Similar to other studies, isoprenaline administration led to an increase in hsTnI, AST, ALT, and LDH [52,53,54,55,56]. Significant mitigation of these effects was accomplished with seven-day PoPEx pre-treatment, thus indicating that pomegranate helps to maintain membrane integrity, therefore restricting the leakage of intracellular enzymes. A previous study by Priscilla et al. (2009) investigated the cardioprotective potential of gallic acid, one of the phenolic acids found in the pomegranate extract [40]. The authors found that 10-day oral pre-treatment with 15 mg/kg of gallic acid led to a significant decrease in CK, CK-MB, AST, ALT, and LDH plasma levels, showing the preservation of membrane integrity. Membrane preservation has also been found by other authors who investigated different antioxidative compounds [42,57], which suggests that PoPEx, through its antioxidative components, prevents oxidative damage of the myocyte cellular membrane, therefore leading to the reduction of isoprenaline-induced myocardial tissue damage and, later on, the preservation of myocardial function. 

To further investigate the extent of myocardial damage, a pathohistological (PH) examination was conducted. Isoprenaline induced severe heart damage with fragmented and degenerated cardiomyocytes, interstitial oedema, and dense inflammatory infiltrate. However, in the PoPEx-pre-treated (P + I) group, no degenerative cardiomyocyte changes were found, but only a slight degree of inflammation and haemorrhage. A similar finding was obtained in the rats pre-treated with gallic acid [40], a phenolic acid that is one of the main components of PoPEx. This was objectified by determining an average myocardial damage score. PoPEx pre-treatment significantly lowered the damage score, further supporting its anti-inflammatory and cardioprotective effects.

Balanced lipid metabolism is an important component of maintaining cardiovascular health. High levels of cholesterol and LDL cholesterol are known predictive factors of potential heart disease that positively correlate with the incidence of AMI. Isoprenaline is a synthetic non-selective β adrenergic agonist that activates adenylate cyclase, leading to an increase in cAMP formation. Subsequently, cAMP-dependent protein kinase A is activated, which further leads to triacylglycerol hydrolysis and hyperlipidaemia [58]. Lipids also play an important role in maintaining stability and modifying the composition of the cellular membrane [42]. HDL, on the other hand, inhibits LDL uptake and facilitates cholesterol transport and catabolism and is therefore in negative correlation with AMI incidence [42]. To analyse the lipid profile, the levels of TC, LDL, HDL, and TG were measured. In the isoprenaline vehicle control group, the results showed a tendency toward an increase in TG, TC, and LDL levels and a decrease in HDL levels, which was consistent with previous studies [41,42]. PoPEx pre-treatment caused a reversal of these effects, but without statistical significance. In a double-blind, placebo-controlled randomised trial, Grabez et al. (2019) showed a similar effect in patients with type 2 DM treated with 500 mg/day of PoPEx over 8 weeks [23]. Using the same extract as in the present study, they noted a significant increase in HDL accompanied by a decline in the plasma levels of LDL, TG, and TC [22]. Although some studies are in agreement with these results [59,60] and others are not [61,62], these discrepancies could be explained by the differences in the experimental protocols. Previous studies that investigated the effects of pomegranate components indicated that polyphenols, mainly punicalin and ellagic acid, showed dose-dependent lipid-lowering effects. The possible molecular mechanisms of these effects are related to the activation of peroxisome proliferator-activated receptor 23 γ (PPARγ) and enhanced cholesterol metabolism [63]. Gallic acid has been reported to inhibit cholesterol esterase, but orally administered polyphenols have also been shown to bind bile acids in the digestive system and therefore increase their faecal excretion [64].

In the I group, an increase in homocysteine (Hcy) levels was noted. Similar results have been found in other studies using the ISO model [65,66,67]. Homocysteine is considered to be an independent risk factor for cardiovascular diseases [68] that increases ROS production, thus causing mitochondrial dysfunction and promoting oxidative damage [69,70,71]. PoPEx administration significantly lowered Hcy levels, which is in concordance with a study by Kannan et al. (2011) [65] who pre-treated rats with ellagic acid, one of the most abundant polyphenols present in the PoPEx extract used in this study.

## 5. Conclusions

The present study provides an initial insight into the use of pomegranate peel extract or PoPEx as an efficient cardioprotective agent in the model of Takotsubo cardiomyopathy. PoPEx administration led to the alleviation of oxidative heart tissue damage, reduced the extent of tissue inflammation, and induced a rise in the antioxidant potential of the myocardium. Nonetheless, further studies are needed to investigate the molecular mechanisms related to the antioxidative and cardioprotective effects of PoPEx in Takotsubo-like cardiomyopathy.

## Figures and Tables

**Table 1:** Effect of PoPEx pre-treatment on biochemical parameters and serum cardiac markers.

	C	I	P	P + I
AST (U/L)	278.60 ± 82.13	1472.17 ± 708.62 *	225.17 ± 58.20	600.57 ± 757.43 #
ALT (U/L)	116.20 ± 41.57	1132.00 ± 1182.71 *	87.17 ± 36.87	192.71 ± 122.08 #
LDH (U/L)	1162.80 ± 545.44	3922.67 ± 1243.23 *	981.50 ± 347.02	1176.33 ± 413.99 #
hsTnI (pg/mL)	70.73 ± 53.24	46,021.13 ± 29,975.57 *	32.40 ± 24.04	888.30 ± 549.43 #
Hcy (µmol/L)	5.24 ± 0.72	15.67 ± 1.67 *	5.88 ± 0.87	11.43 ± 1.72 #

All values are expressed as mean ± SEM. C—control group; P—pomegranate control group; I—isoprenaline group; P + I—pomegranate + isoprenaline group. AST—aspartate transaminase; ALT—alanine transaminase; LDH—lactate dehydrogenase; hsTnI—high-sensitive troponin Hcy—homocysteine; * *p* < 0.05 vs. C; # *p* < 0.05 vs. I.

**Table 2:** Effect of PoPEx pre-treatment on lipid status.

	C	I	P	P + I
TC (mmol/L)	1.24 ± 0.15	1.37 ± 0.20	1.02 ± 0.12	1.21 ± 0.31
HDL (mmol/L)	0.58 ± 0.08	0.45 ± 0.08	0.45 ± 0.08	0.49 ± 0.21
LDL (mmol/L)	0.10 ± 0.00	0.22 ± 0.04	0.10 ± 0.00	0.19 ± 0.07
TG (mmol/L)	1.42 ± 0.42	1.83 ± 1.78	1.43 ± 0.47	1.19 ± 0.32

All values are expressed as mean ± SEM. C—control group; P—pomegranate control group; I—isoprenaline group; P + I—pomegranate + isoprenaline group. TC—total cholesterol; HDL—high-density lipoproteins; LDL—low-density lipoproteins; TG—triglycerides. Statistical analysis was done using Tukey and Bonferroni tests for post hoc analysis and no statistical significance was found (*p* > 0.05).

## Data Availability

The data presented in this study are available on request from the corresponding author.
